# The Curvilinear Relationship Between Career Calling and Work Fatigue: A Moderated Mediating Model

**DOI:** 10.3389/fpsyg.2020.583604

**Published:** 2020-10-30

**Authors:** Jie Zhou, Jian wei Zhang, Xing yu Xuan

**Affiliations:** ^1^Department of Police Management, Sichuan Police College, Luzhou, China; ^2^School of Humanities and Social Sciences, Beijing Institute of Technology, Beijing, China

**Keywords:** career calling, work fatigue, role overload, COVID-19 event disruption, curvilinear relationship

## Abstract

Drawing on the job demands-resources (JD-R) model and event system theory (EST), this study constructed a moderated mediating model to investigate the direct effect of career calling on work fatigue, the mediating effect of role overload, and the moderating effect of COVID-19 event disruption in the above relationships. We administered an online questionnaire to 488 Chinese police officers who participated in frontline work to prevent and control the COVID-19 pandemic. The results showed a U-shaped curvilinear relationship of career calling with physical fatigue, mental fatigue, emotional fatigue, and role overload. Moreover, role overload partially mediated these curvilinear relationships. In addition, COVID-19 event disruption positively moderated the direct curvilinear effect of career calling on role overload, physical fatigue, and emotional fatigue, as well as the first stage of the mediating effect in the relationship between career calling and physical, mental, and emotional fatigue through role overload. Furthermore, the direct U-shaped curvilinear effects and the indirect effects were more significant when COVID-19 event disruption was high.

## Introduction

Over the past decade, career calling has been a hot topic in the occupational psychology and organizational behavior fields (Elangovan et al., 2010; Dobrow, 2013; Duffy and Dik, 2013; Zhang et al., 2014, 2015) because of its association with positive individual outcomes, such as career success, work engagement (Xie et al., 2016), satisfaction (Hagmaier and Abele, 2012; Hirschi and Herrmann, 2012; Duffy et al., 2013, 2014; Douglass et al., 2015; Chen et al., 2016), well-being (Duffy and Dik, 2013), job performance (Park et al., 2015; Kim et al., 2018), voice behavior, and organizational citizenship behavior (Xie et al., 2017; Park et al., 2018). However, in recent years, the dark side of career calling has also received increasing attention from scholars and practitioners (Zhang et al., 2017; Duffy et al., 2018; Lysova et al., 2018, 2019; Michaelson and Tosti-Kharas, 2019; Sturges et al., 2019). For example, studies have found that an increased level of career calling was positively linked to negative workplace outcomes, such as excessive workload, workaholic behavior, job burnout, and fatigue (Cardador and Caza, 2012; Hirschi and Herrmann, 2012; Dobrow, 2013; Duffy and Dik, 2013; Berkelaar and Buzzanell, 2015; Duffy et al., 2016, 2018; Keller et al., 2016; Zhao et al., 2016; Clinton et al., 2017; Hirschi et al., 2019).

Nevertheless, knowledge on the potential non-linear effect of career calling on workplace outcomes is limited. As mentioned above, even though substantial progress has been made in the career calling field, including growing research on its positive, negative, and double-edged-sword effects, most of these have been explored as linear relationships, and its potential curvilinear effect has not yet received equivalent attention. In the context of growing research on career calling, it is important to investigate whether there is variation in the influence of different levels of career calling on workplace outcomes (Thompson and Bunderson, 2019), rather than simply discussing its effects under the conditions of low and high level. This may help to better understand the double-edged-sword effect of career calling on workplace outcomes. Hence, it is important to further explore the potential curvilinear effect of career calling on detrimental workplace outcomes (Duffy et al., 2018; Lysova et al., 2019), such as work fatigue.

In addition, much less is known about whether the strength, intensity, or character of the career calling experience are related to specific occupations, and if so, why? (Thompson and Bunderson, 2019). Compared with other occupations in which the societal contributions of the career are less obvious or the skills required are less distinctive, career calling may be more common in police settings, where the sacrifice for clear public benefits (Bunderson and Thompson, 2009) and the need for distinct skills lend police officers a sense of outer requirement as well as inner passion and talent (Thompson and Bunderson, 2019). This is particularly relevant in the case of the 2019 coronavirus (COVID-19) pandemic, where police officers must have unique skills and a sense of obligation to keep society and citizens safe. Thus, it must be explored if the pandemic may make them perceive a higher sense of career calling than other occupations.

Moreover, the potential curvilinear effect of career calling on police officers’ work fatigue, and the mechanism accounting for this relationship in the situation of COVID-19 pandemic needs to be further investigated. It is known that the COVID-19 pandemic as a situational factor has an overt effect on police officers’ physical and mental health at work (Zhu et al., 2020). Work fatigue and role overload are typical detrimental workplace outcomes (Laufs and Waseem, 2020; Zhu et al., 2020). Particularly in recent years, many police officers, experiencing an exaggerated level of career calling from the social responsibility and inherent meaning associated with their work, have suffered serious physical and psychological problems because of excessive work investment, workload, and chronic fatigue (Fekedulegn et al., 2017; Zhu et al., 2020). This is a practical issue at police departments that needs to be addressed.

**FIGURE 1:**
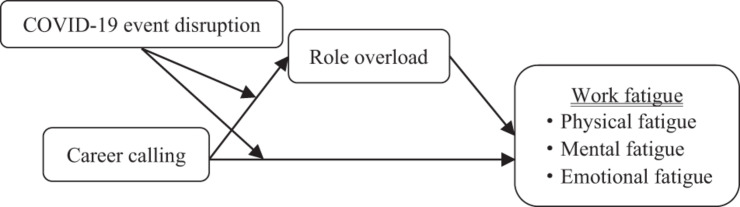
The theoretical model.

The current study aimed to examine whether and how career calling shows a curvilinear relationship with work fatigue among police officers under the COVID-19 pandemic conditions. To shed light on this question, we conducted a moderated mediating model based on the job demands-resources (JD-R) model (Demerouti et al., 2001) and event system theory (EST) (Morgeson et al., 2015) to test the possible curvilinear relationship between career calling and (a) work fatigue, (b) the mediating effect of role overload in the direct relationship, and (c) the moderating effect of COVID-19 event disruption on the direct effect as well as the first stage of the mediating effect. By addressing these issues, this study aimed to contribute to advancing the theoretical construct of career calling and providing a better understanding of how career calling can simultaneously decrease and increase work fatigue in a curvilinear way under the COVID-19 pandemic disruption. Additionally, the study highlights the importance of paying more attention to the negative side and the curvilinear effect of career calling in practice management, particularly for emergency workers. The theoretical model is shown in Figure 1.

## Theory and Hypothesis Development

### JD-R Model and EST

The JD-R model classifies the work environment characteristics in job demands and job resources (Demerouti et al., 2001). The former refers to the physical, social, or organizational aspects of the job that require sustained physical or psychological effort and are associated with relevant costs. The latter denotes the physical, psychological, social, or organizational aspects of the job that may be functional in achieving work goals, reduce job demands with associated physiological and psychological costs, and stimulate personal growth and development. The fit between job demands and personal resources will produce a positive effect on work outcomes, while an imbalance will lead to a negative effect. Career calling is an important job-related personal resource (Creed et al., 2014; Praskova et al., 2014). When balanced with job demands, it can stimulate individuals to achieve career goals, career development, and success (Hall and Chandler, 2005), as well as reduce job burnout (Hagmaier and Abele, 2012; Deng and Chen, 2019) and work fatigue (Frone and Tidwell, 2015). However, exaggerated job demands could lead individuals to excessively invest themselves in their work (Keller et al., 2016), in turn leading to work fatigue from depleted job-related personal resources (Fekedulegn et al., 2017; Hirschi et al., 2019). The JD-R model is often used to explain the influence mechanism of job burnout (Demerouti et al., 2001). Given that burnout is highly correlated with work fatigue, we suggest that this model could also provide a theoretical basis for examining the curvilinear mechanism underlying the relationship between career calling and work fatigue.

The EST poses that an event has direct and indirect effects on individuals’ psychological and behavioral patterns from top to bottom, and classifies the event strength according to event criticality, novelty, and disruption. EST incorporates context to theorize and provide an insightful way to quantify events’ effects (Morgeson et al., 2015; Johns, 2017). It has been widely applied to research on emotions and attitudes, such as firefighters’ emotional problems following 9/11 (Bacharach and Bamberger, 2007) and the COVID-19 pandemic factors affecting Chinese people’s social participating willingness (Zheng, 2020). The COVID-19 pandemic is a disruptive event that, worldwide, has greatly disrupted police officers’ normal way of work and life. The existing theories on career calling and work fatigue cannot comprehensively interpret the disruptive effect of the COVID-19 pandemic in relation to police officers’ career calling and work fatigue. Moreover, the COVID-19 pandemic is a major public health emergency with the fastest spread, the widest infection range, and the most difficult prevention and control requirements since the founding of the People’s Republic of China, which exactly matches the concept of event disruption. Hence, to further explore contextual factors in the positive and negative effects of career calling on diverse outcomes (Cohen and Duberley, 2015), EST is suitable for identifying the relationship between career calling and work fatigue in the context of the COVID-19 pandemic disruption.

### Hypothesis Development

#### The Curvilinear Relationship Between Career Calling and Work Fatigue

To date, there is no consensus on the definition of career calling. The controversy has focused on the source (external or internal drive) (Hall and Chandler, 2005; Elangovan et al., 2010; Dobrow and Tosti-Kharas, 2011; Hirschi, 2011) and orientation (specific or general occupations) (Steger et al., 2010; Dobrow and Tosti-Kharas, 2011). Nevertheless, Duffy et al. (2018) integrated previous definitions and redefined career calling as an approach to work that reflects seeking a sense of overall purpose and meaning and is used to help others or contribute to the common good, motivated by an external or internal summons. Work fatigue refers to individuals experiencing extreme physical, mental, and emotional tiredness and dysfunction, which reduces their capacity to engage in activities during and at the end of the workday (Frone and Tidwell, 2015). Generally, police officers experience a higher level of career calling and experience more work fatigue than the general population (Fekedulegn et al., 2017; Zhu et al., 2020), as they work in a chronically unpredictable, dangerous, changing, and increasingly stressful environment.

According to the JD-R model (Demerouti et al., 2001), career calling is a positive job-related personal resource (Xanthopoulou et al., 2009; Creed et al., 2014; Praskova et al., 2014; Lysova et al., 2019), and decreasing, increasing, or depleting this resource can lead to diverse outcomes. Primary research found that a low or decreased level of career calling and insufficient or loss of job-related personal resources could result in consumption of other resources and lead to subsequent work fatigue (Clinton et al., 2017). Moreover, a higher level of career calling was linked with more initial job-related personal resources and positively correlated with work passion, work and life meaning, and satisfaction (Duffy et al., 2012; Hagmaier and Abele, 2012; Neubert and Halbesleben, 2015). Furthermore, a higher level of career calling helps buffer physical, mental, and emotional fatigue caused by work stress, emotional exhaustion (Wright and Cropanzano, 2000; Penney et al., 2011), and job burnout (Hagmaier and Abele, 2012; Deng and Chen, 2019).

Nevertheless, despite career calling being a positive psychological construct, extremely high levels might not be desirable. This is because individuals with an exaggerated level of career calling have excess mental and emotional resources, which may lead to excessive levels of sacrifice for their work, workload, career commitment, and investment even under time constraints, culminating in a workaholic state (Dik and Duffy, 2012; Keller et al., 2016; Xie et al., 2016; Duffy et al., 2018; Hirschi et al., 2019). Moreover, long work hours, heavy workloads (Elangovan et al., 2010; Aronsson et al., 2017; Fekedulegn et al., 2017), increased work–life conflicts, job burnout (Bunderson and Thompson, 2009), and insufficient sleep (Clinton et al., 2017) will ultimately lead to increased work fatigue (Berkelaar and Buzzanell, 2015; Frone and Tidwell, 2015) by depleting job-related personal resources. In contrast, a moderate level of career calling could balance job demands and related personal resources without excessive resource depletion, improve individuals’ work status, and further reduce work fatigue. In short, as police officers’ career calling increases, their work fatigue follows a process of decreasing to optimum levels and then increases. Thus, there is a U-shaped curvilinear relationship between career calling and work fatigue. Accordingly, we hypothesized the following:

H1: Career calling has a U-shaped curvilinear relationship with physical fatigue (1a), mental fatigue (1b), and emotional fatigue (1c).

### The Curvilinear Relationship Between Career Calling and Role Overload

Role overload refers to a role-related stressor that individuals experience when they lack the necessary time and resources to complete role demands (Coverman, 1989; Örtqvist and Wincent, 2006). It is prevalent among police officers, as they are often required to undertake many social responsibilities and play several roles at work (Duxbury et al., 2015), such as maintaining social stability, detecting criminal cases, and preventing adverse events such as policy violations. Role overload is often associated with individuals’ resources (Peterson et al., 1995; Li and Wang, 2018), such as excessive work passion, commitment, and load caused by an exaggerated level of career calling.

The JD-R model implies that individuals with a decreased level of career calling have insufficient physical and psychological resources to cope with job demands caused by role overload (Demerouti et al., 2001; Creed et al., 2014). In situations of higher job demands and lower resources, individuals demonstrate less self-efficacy to solve problems with limited job-related personal resources and are more likely to experience role overload (Dasgupta, 2012). Therefore, a lower level of career calling does not help to reduce role overload. In addition, by increasing individuals’ level of career calling to a moderate level, accompanied by growing job-related personal resources, individuals will experience a lower role overload, as job loads and roles will fit job-related personal resources.

However, the positive effect of career calling on role overload will also probably change to negative as an optimal point is surpassed. Specifically, when individuals’ level of career calling continuously increases and becomes excessive, job demands such as job role and workload increase simultaneously. In this situation, individuals with an exaggerated level of career calling have excessive responsibility and commitment to work and will try their best to undertake more roles and tasks, even when their resources are limited (Daskin, 2016). Thus, work overload keeps individuals in a heavy work situation (Mainemelis, 2001), and multiple roles increase the demand for resources. Under this condition, unbalanced job demands and personal resources will lead to increased role overload (Suarthana and Riana, 2016; Qureshi et al., 2019), especially for individuals with an excessive level of career calling in collectivist cultures (Peterson et al., 1995). In summary, both a lower and an exaggerated level of career calling, causing imbalance between job demands and related personal resources, can lead to role overload; thus, the most appropriate is a moderate level of career calling. Accordingly, we hypothesized the following:

H2: Career calling has a U-shaped curvilinear relationship with role overload.

### The Mediating Effect of Role Overload

The unexpected outbreak of the COVID-19 pandemic has dramatically disrupted the pace of life and work and has caused a wide range physical and psychological stress in individuals, particularly for the frontline workers (Cunill et al., 2020; Xiang et al., 2020). Teng et al. (2020) showed that most participants felt fatigue (73.7%) and depression (50.0%) among 2614 Chinese frontline workers after the COVID-19 pandemic outbreak. Role overload, a typical role stressor, is a key outcome of resource loss and is positively linked to work fatigue (Frone and Tidwell, 2015) and job burnout (Posig and Kickul, 2003; Surana and Singh, 2013; De Beer et al., 2015; Vullinghs et al., 2018). Based on the JD-R model, police officers experiencing overstrain and overwork will consume their stock of job-related resources, both physiological and psychological (Bowling et al., 2015). Insufficient resources stock for the job demands will lead to work fatigue (Bin, 2008). As such, after the COVID-19 pandemic breakout, the pandemic itself and civilians’ despondence, fear, and anxiety under quarantine measures (Brooks et al., 2020) may increase police officers’ workload and stressors. Thus, they have to adopt more roles to cope with the workload and stressors (Hecht, 2001), which may consume their insufficient stock of job-related personal resources (Duxbury et al., 2015). Next, with a continuously growing role overload, more resources will be lost, deviating from their normal function (Bin, 2008); this may consequently result in severe physical (Nixon et al., 2011), mental, and emotional fatigue (Kristie, 2017), for example, depression, anxiety, and insomnia (Teng et al., 2020).

In addition, according to Hypotheses 1 and 2, we expect a possible U-shaped curvilinear influence path from career calling to work fatigue through role overload. Because a moderate level of career calling balances out job demands and personal resources, such individuals will not experience much role overload; thus, less work fatigue will be experienced. However, a lower or an exaggerated level of career calling will have the opposite effect on police officers’ work fatigue via role overload. Specifically, when police officers have a lower level of career calling, they will experience role overload, as job-related resources will be insufficient to cope with the demands of multiple roles, which, in turn, will increase work fatigue. Besides, when police officers’ level of career calling is exaggerated, although they have enough resources, they are more likely to undertake tasks and partake in more job-related roles, given their exaggerated intrinsic drives and external summons; moreover, they will become overly immersed and overinvolved in tasks. This will cause depletion of physiological and psychological resources and inability to cope with job demands, which will result in work fatigue. Accordingly, we proposed the following:

H3: Role overload plays a mediating role in the U-shaped curvilinear relationship of career calling with physical fatigue (3a), mental fatigue (3b), and emotional fatigue (3c).

### The Moderating Effect of COVID-19 Event Disruption

Event disruption refers to individuals being controlled and disrupted by a destructive event and having to adjust their psychological state and behavioral patterns in response (Morgeson et al., 2015). This study considers the COVID-19 pandemic as such an event. Previous research demonstrated that events can have an important impact on individuals’ workplace outcomes (Duffy et al., 2016; Van Iddekinge et al., 2018). During the COVID-19 pandemic, most police officers have been experiencing increased work roles and workload, which in turn increases their work fatigue (Stogner et al., 2020). For example, Zhu et al. (2020) surveyed 5,467 Chinese police officers in Hubei province after outbreak and found that more than 70% were stressed by workload and role overload. Recently, scholars found that COVID-19 event disruption influences and differs in terms of individuals’ activity engagement from a macro level (Zheng, 2020). Thus, the COVID-19 pandemic as a typical contextual factor may also act as a boundary condition in the U-shaped curvilinear effect of career calling on both role overload and work fatigue.

To be more specific, we firstly propose that a higher level of COVID-19 event disruption strengthens the curvilinear relationship between career calling and role overload. Based on the EST (Morgeson et al., 2015) and JD-R model (Demerouti et al., 2001), when the COVID-19 pandemic is highly disruptive, the normal social order, civilians’ life and work, as well as police officers’ job plans and executive ability are more seriously disrupted. Thereby, more job demands, such as emergent tasks and work roles, need to be undertaken and more job-related resources are required. In such instances, for those police officers having a lower level of career calling, insufficient job-related personal resources cannot meet the excessive job demands, which will cause severe role overload. For officers having an exaggerated level of career calling, strong external summons and internal obligation will lead them to devote themselves to more job roles and higher workloads, and such excessive job demands and depletion of resources will finally lead to role overload. For officers with a moderate level of career calling, the balance between job demands and personal resources will prevent role overload. Conversely, when the COVID-19 pandemic is less disruptive, that is, the situational stimulus is weak, the curvilinear relationship between career calling and role overload will not be significantly changed, since a low level of event disruption will not alter police officers’ usual job plans. Accordingly, we proposed the following:

Hypothesis 4: COVID-19 event disruption significantly moderates the U-shaped curvilinear relationship between career calling and role overload, such that the U-shaped curvilinear relationship is stronger when COVID-19 event disruption is high.

In addition, by the same token, we propose that a higher level of COVID-19 event disruption strengthens the curvilinear relationship between career calling and work fatigue. Specifically, when the COVID-19 pandemic is highly disruptive, police officers’ life and work change, and job demands increase. Under this condition, police officers with a lower level of career calling, which means fewer job-related resources, cannot meet the excessive job demands. Hence, officers have to sacrifice other physical, mental, and emotional resources to complete job tasks, which, in turn, causes serious work stress and job burnout (Bunderson and Thompson, 2009; Duffy et al., 2018; Hirschi et al., 2019). Finally, the imbalance between resources and demands will result in symptoms such as physical, mental, and emotional fatigue (Frone and Tidwell, 2015; Duffy et al., 2016; Keller et al., 2016). Those officers with an exaggerated level of career calling, stemming from a sense of social responsibility and the inherent meaning associated with their work, will overcommit themselves to increasing job demands, unable to detach themselves from the excessive workload, given the greater social and public benefit associated with their work (Thompson and Bunderson, 2019). In such workaholic state, job-related personal resources will be depleted, and other resources will be sacrificed under excessive job demands. Consequently, this will lead to more physical, mental, and emotional fatigue (Frone and Tidwell, 2015; Duffy et al., 2016; Keller et al., 2016). For those officers with a moderate level of career calling, the balance between job demands and personal resources will prevent work fatigue. In contrast, when the COVID-19 pandemic is less disruptive, which means that the situational stimulus is weak, the balance between job demands and personal resources does not fluctuate dramatically; hence, the curvilinear relationship may not change significantly. Accordingly, we proposed the following:

Hypothesis 5: COVID-19 event disruption significantly moderates the U-shaped curvilinear relationship between career calling and physical fatigue (5a), mental fatigue (5b), and emotional fatigue (5c), such that the U-shaped curvilinear relationship is stronger when COVID-19 event disruption is high.

According to Hypotheses 3, 4 and 5, we further propose that COVID-19 event disruption will not only moderate the direct effect of career calling on role overload and work fatigue but also moderate the mediating effect of role overload between career calling and work fatigue. When COVID-19 event disruption is high, both a lower and an exaggerated level of career calling will cause an imbalance between job demands and job-related personal resources, increase role overload, and in turn induce work fatigue. Moreover, a moderate level of career calling will have the strongest restraining effect on work fatigue through role overload, without increasing excessive job demands and depleting resources. However, this moderating effect will not be significant when COVID-19 event disruption is low. Therefore, we proposed a moderated mediating hypothesis, that is, the COVID-19 event disruption moderates the mediating effect of role overload in the U-shaped curvilinear relationship between career calling and work fatigue.

Hypothesis 6: COVID-19 event disruption significantly moderates the U-shaped curvilinear relationship between career calling and physical fatigue (6a), mental fatigue (6b), and emotional fatigue (6c) through the mediating effect of role overload, and the mediating effects are stronger when COVID-19 event disruption is high.

## Materials and Methods

### Participants and Procedure

The isolation policy prevented conducting a field study during the COVID-19 pandemic from February to April 2020. Therefore, we conducted an online questionnaire. A total of 512 questionnaires were distributed and returned. After excluding 24 invalid questionnaires with remarkable consistency, too long (more than 5 min), or too short (less than 1 min) response time, 488 valid questionnaires were analyzed, for a valid response rate of 95.31%. Males accounted for 79.5% (388) and females accounted for 20.5% (100) of the sample. Age 30 and below were 30.9% (151), 52.9% (258) were age 31–40, 9.7% (47) were age 41–50, and 6.6% (32) were age 51 and older. Police service time of less than 1 year accounted for 10.2% (50), 1–10 years accounted for 50.4% (246), 11–20 years accounted for 27.9% (136), and more than 20 years accounted for 11.5% (56). College degree and below accounted for 28.1% (137) while bachelor’s degree and above accounted for 71.9% (351).

### Measures

It should be noted that the COVID-19 pandemic outbreak coincided with the Chinese New Year, when police officers’ workloads are substantially increased. To reduce questionnaire response time as much as possible to ensure reliability and validity of all measurements, we selected typical items from the mature scales with a higher factor loading. We performed a confirmatory factor analysis (CFA) using Amos 24.0 to test construct validity for the shortened scales. In addition, for the English language scales, we strictly followed a translation and back-translation procedure (Brislin, 1980).

### Career Calling

We used the Chinese version of a 12-item career calling questionnaire developed by Dobrow and Tosti-Kharas (2011) and translated into Chinese by Pei and Zhao (2015) (α = 0.94). Four items with the highest factor loading and a Likert five-point scale (1 = strongly disagree, 5 = strongly agree) were adopted. An example item was “Being a policeman/policewoman is a deeply moving and gratifying experience for me.” In this study, α was 0.76, and the CFA results showed that the single-dimensional model fit was good (χ^2^/df = 2.664, RMSEA = 0.058, GFI = 0.994, TLI = 0.985, CFI = 0.995).

### Work Fatigue

We applied the work fatigue scale developed by Frone and Tidwell (2015), which measures physical fatigue (α = 0.94), mental fatigue (α = 0.95), and emotional fatigue (α = 0.96), with six items for each measure. Three items with the highest factor loading of each dimension were adopted for our study. Each used a five-point Likert scale from 1 (never) to 5 (everyday). A sample item for physical fatigue was “After COVID-19 breaking out, how often did you feel physically exhausted at the end of the workday?” The α for physical fatigue was 0.88. A sample item for mental fatigue was “After COVID-19 breaking out, how often did you feel mentally exhausted at the end of the workday?” The α for mental fatigue was 0.88. An example item for emotional fatigue was “After COVID-19 breaking out, how often did you feel emotionally worn out at the end of the workday?” The α for emotional fatigue was 0.88. In this study, the α for the full version of work fatigue was 0.95, and the CFA results showed that the three-dimensional model was a good fit (χ^2^/df = 2.492, RMSEA = 0.055, GFI = 0.981, TLI = 0.987, CFI = 0.994).

### Role Overload

We used a three-item role overload scale that measures time pressure and workload, developed by Schaubroeck et al. (1989) (α = 0.75). This scale showed good reliability when used to measure frontline employees’ role overload in China (Ding and Yu, 2020) (α = 0.88) and the United States (Bolino and Turnley, 2005) (α = 0.80). Three items included “I have too much work to do everything well,” “The amount of work I am asked to do is unfair,” and “I never seem to have enough time to get everything done.” In the current study, the α for role overload was 0.94.

### COVID-19 Event Disruption

We used a four-item event disruption scale developed by Morgeson (2005) and Morgeson and DeRue (2006) and translated into Chinese by Liu and Liu (2017). This scale has been widely used by scholars and has shown acceptable reliability. For example, Chen et al. (2020) measured workplace event disruption in China and reported α = 0.75. Zheng (2020) measured the COVID-19 event disruption in a sample of 411 Chinese people and reported an α of 0.74. Two sample items were: “The COVID-19 event alters your normal way of responding,” and “The COVID-19 event disrupts your ability to get your work done.” In this study, the value of α for COVID-19 event disruption was 0.72.

### Organizational Support

We used a three-item organizational support scale from Eisenberger et al. (1986), which has been widely used in China and has shown good reliability and validity. Three items were “The organization is willing to help me when I need a special favor,” “The organization cares about my opinions,” and “The organization really cares about my well-being.” In this study, α for organizational support was 0.83.

### Control Variables

Previous studies revealed that work fatigue (Ricci et al., 2007; Rose et al., 2017; Deng and Chen, 2019) is affected by demographic variables, and organizational support is significantly correlated with career calling (Duffy et al., 2018; Presbitero and Teng-Calleja, 2019), role overload (Kim et al., 2019), and work fatigue (Qiu et al., 2020). Therefore, we controlled for gender (male = 0, female = 1), education (below bachelor’s = 0, bachelor’s and above = 1), age, police tenure, and organizational support.

## Results

### Preliminary Analyses

#### Common Method Bias Test and Discriminant Validity Test

The study used a cross-sectional design and the data were self-reported; therefore, we tested for common method bias using the single factor method and partial correlation analysis, respectively (Podsakoff et al., 2003). The CFA results showed that the single factor model had a poor fit (χ^2^/df = 14.136, RMSEA = 0.164, GFI = 0.647, TLI = 0.653, CFI = 0.702). In addition, we regarded organizational support as a measured markable variable and ran a partial correlation analysis. The results showed that organizational support was significantly positively correlated with career calling (*r* = 0.439, *p* < 0.001), and negatively correlated with work fatigue (*r* = −0.195, *p* < 0.001) and role overload (*r* = −0.252, *p* < 0.001), but uncorrelated with COVID-19 event disruption (*r* = 0.074, *p* > 0.05). In addition, after controlling for organizational support, compared with the zero-order correlations, the partial correlations between work fatigue and career calling (*r*_*zero order*_ = −0.195, *p* < 0.001; *r*_*partial*_ = −0.158, *p* < 0.001), role overload (*r*_*zero order*_ = 0.720, *p* < 0.001; *r*_*partial*_ = 0.716, *p* < 0.001), and COVID-19 event disruption (*r*_*zero order*_ = 0.231, *p* < 0.001; *r*_*partial*_ = 0.237, *p* < 0.001) were still significant. Accordingly, this study demonstrated some common method bias, but it was not serious.

Moreover, we tested the discriminant validity with CFA. The results showed that the research model was a better fit (χ^2^/df = 3.810, RMSEA = 0.076, GFI = 0.884, TLI = 0.926, CFI = 0.938) than other models. Therefore, the discriminant validity was acceptable.

#### Descriptive Analysis

We used IBM SPSS Statistics 25.0 to conduct the descriptive analysis. Table 1 presents the means, standard deviations, and correlations of all variables. The results showed that career calling was negatively and significantly correlated with physical fatigue, mental fatigue, emotional fatigue, and role overload, but was not significantly correlated with COVID-19 event disruption. The coefficients between variables ranged from −0.272 to 0.854. The results were in accordance with our hypotheses and were suitable for further hypothesis testing.

**TABLE 1:** Means, standard deviations, and correlation coefficients of variables.

Variables	*M*	*SD*	1	2	3	4	5
(1) Career calling	3.607	0.833	—				
(2) Physical fatigue	3.664	0.976	−0.109^∗^	—			
(3) Mental fatigue	3.372	1.021	−0.203^∗∗∗^	0.845^∗∗∗^	—		
(4) Emotional fatigue	3.023	1.022	−0.272^∗∗∗^	0.692^****^	0.854^∗∗∗^	—	
(5) Role overload	3.361	0.981	−0.252^∗∗∗^	0.600^∗∗∗^	0.715^∗∗∗^	0.700^∗∗∗^	—
(6) COVID-19 event disruption	3.663	0.696	0.074	0.239^∗∗∗^	0.206^∗∗∗^	0.156^∗∗∗^	0.178^∗∗∗^

*p < 0.05, **p < 0.01, ***p < 0.001.

### Hypothesis Test

We used the PROCESS macro from Hayes (2013) to test our hypotheses. In the model templates, Model 8 assumed that the moderator W (COVID-19 event disruption) moderated the direct effect (career calling → work fatigue) and the first stage of the mediating effect (career calling → role overload → work fatigue), which was consistent with our theoretical model. In addition, all variables were standardized during the analysis and we controlled for demographic variables and organizational support.

As shown in Table 2, in Models 3, 6, and 9, when controlling for demographic variables, organizational support, and the linear term effect of career calling, the quadratic term of career calling positively predicted physical fatigue (β = 0.180, *p* < 0.001), mental fatigue (β = 0.246, *p* < 0.001), and emotional fatigue (β = 0.164, *p* < 0.01), indicating a U-shaped curvilinear relationship between career calling and physical, mental, and emotional fatigue, supporting Hypotheses 1a to 1c.

**TABLE 2:** The moderated mediating effect analysis.

Variables	Role overload		Physical fatigue			Mental fatigue			Emotional fatigue		
	M1	M2	M3	M4	M5	M6	M7	M8	M9	M10	M11
Gender	–0.064	–0.078	–0.010	0.029	0.027	–0.013	0.032	0.036	–0.065	–0.024	–0.022
Age	–0.128	−0.158*	–0.112	–0.034	–0.046	–0.076	0.013	0.016	0.009	0.092	0.092
Tenure	0.034	0.052	0.123	0.102	0.115	0.050	0.026	0.028	–0.027	–0.049	–0.044
Education	–0.029	–0.059	0.012	0.029	–0.002	0.009	0.029	0.013	0.090*	0.109**	0.096**
Organizational support	−0.118*	−0.114*	–0.054	0.018	0.029	−0.125*	–0.043	–0.031	−0.290***	−0.214***	−0.206***
Career calling	−0.225***	−0.266***	–0.075	0.062	0.021	−0.131*	0.024	0.006	−0.118*	–0.029	0.015
Career calling^2^	0.108*	0.075	0.180***	0.114**	0.103**	0.246***	0.171***	0.172***	0.164**	0.094**	0.092**
Role overload				0.607***	0.573***		0.692***	0.677***		0.650***	0.642***
COVID-19 event disruption		0.100			0.021			0.041			–0.035
Career calling × COVID-19 event disruption		0.094			–0.047			−0.074*			–0.049
Career calling^2^ × COVID-19 event disruption		0.138*			0.167**			0.058			0.096*
*R*	0.326	0.397	0.225	0.617	0.644	0.340	0.737	0.746	0.419	0.744	0.750
*R*^2^	0.106	0.158	0.051	0.380	0.415	0.115	0.543	0.557	0.175	0.553	0.562
*F*	8.132***	8.939***	3.655**	36.774***	30.641***	8.950***	71.122***	54.305***	14.568***	74.068***	55.544***

Standardized coefficients were presented. *p < 0.05, **p < 0.01, ***p < 0.001.

Model 1 in Table 2 shows that the quadratic term of career calling significantly and positively predicted role overload (β = 0.108, *p* < 0.05), supporting Hypothesis 2. As shown in Model 4, Model 7, and Model 10 in Table 2, after adding role overload as a mediator, role overload and the quadratic term of career calling still significantly and positively predicted physical fatigue (β = 0.607, *p* < 0.001; β = 0.114, *p* < 0.01), mental fatigue (β = 0.692, *p* < 0.001; β = 0.171, *p* < 0.001), and emotional fatigue (β = 0.650, *p* < 0.001; β = 0.094, *p* < 0.01). This indicated that role overload partially mediated the U-shaped curvilinear relationships, supporting Hypotheses 3a to 3c.

**FIGURE 2:**
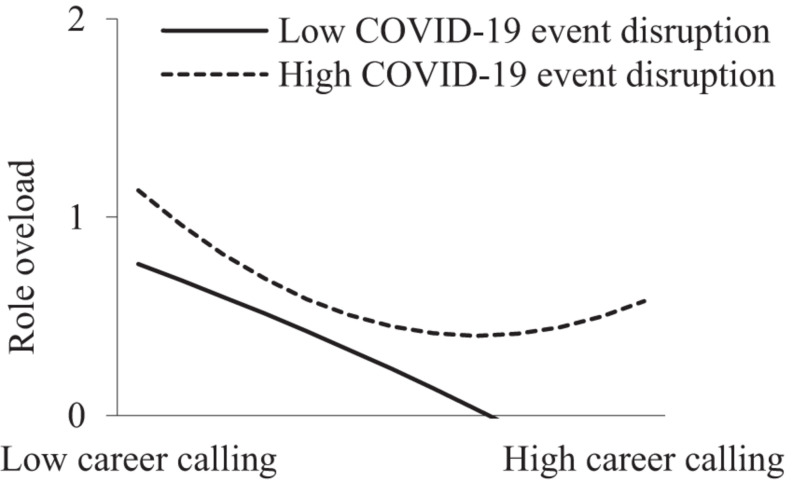
Moderating effect of COVID-19 event disruption between career calling and role overload.

**FIGURE 3:**
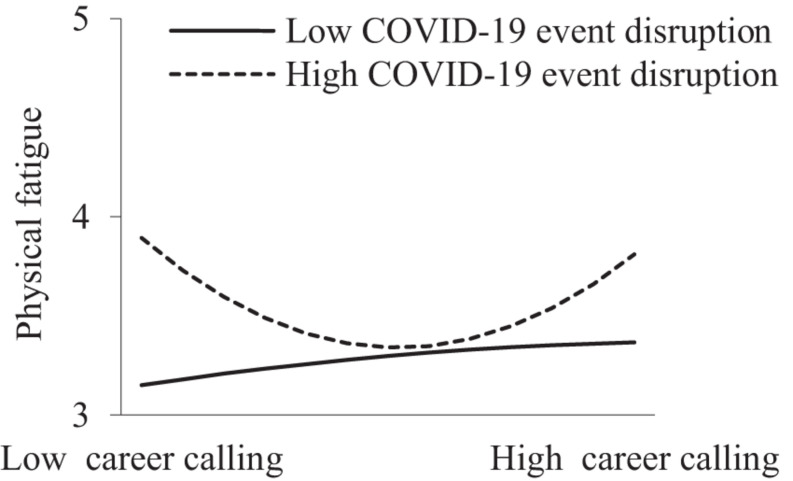
Moderating effect of COVID-19 event disruption between career calling and physical fagitue.

**FIGURE 4:**
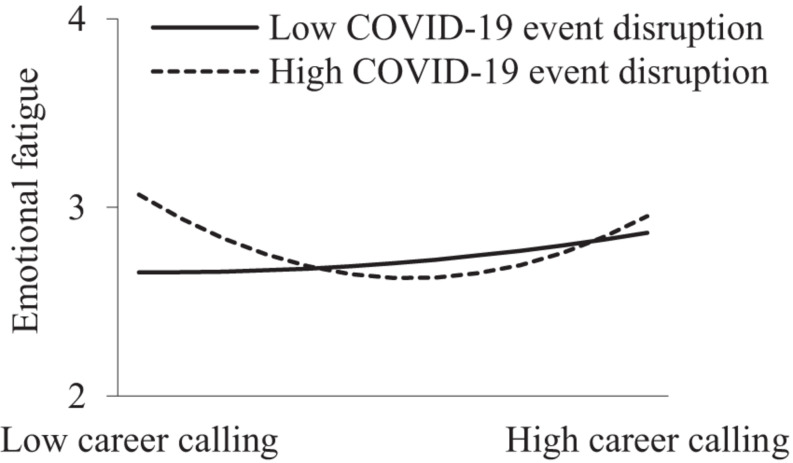
Moderating effect of COVID-19 event disruption between career calling and emotional fagitue.

In addition, as shown in Models 2, 5, 8, and 11 in Table 2, the interactive term of the career calling quadratic term and COVID-19 event disruption significantly and positively predicted role overload (β = 0.138, *p* < 0.05), physical fatigue (β = 0.167, *p* < 0.01), and emotional fatigue (β = 0.096, *p* < 0.05). This suggests that COVID-19 event disruption moderated the curvilinear relationships. However, the effect of the career calling quadratic term on mental fatigue was not significant (β = 0.058, *p* > 0.05). Figures 2–4 show that when COVID-19 event disruption was high, the curvilinear relationships between career calling and role overload (slope_*high*_ = 0.181, *p* < 0.01; slope_*low*_ = −0.025, *p* > 0.05), physical fatigue (slope_*high*_ = 0.227, *p* < 0.001; slope_*low*_ = −0.017, *p* > 0.05), and emotional fatigue (slope_*high*_ = 0.171, *p* < 0.001; slope_*low*_ = 0.024, *p* > 0.05) were more significant, whereas the above relationships were not significant when COVID-19 event disruption was low, supporting Hypotheses 4, 5a, and 5c.

Successively, the results in Table 3 show that all bias-corrected 95% CIs for moderated mediating effects of career calling^2^ → role overload → physical fatigue [*B* = 0.058, 95% CI = 0.014, 0.115], career calling^2^ → role overload → mental fatigue [*B* = 0.071, 95% CI = 0.019, 0.135], and career calling^2^ → role overload → emotional fatigue [*B* = 0.068, 95% CI = 0.017, 0.129] excluded zero. Moreover, when COVID-19 event disruption was high, the bias-corrected 95% CI of the mediating effect [*B* = 0.096, 95% CI = 0.040, 0.159; *B* = 0.118, 95% CI = 0.044, 0.191; *B* = 0.112, 95% CI = 0.045, 0.186] excluded zero, supporting Hypotheses 6a to 6c.

**TABLE 3:** The bootstrap test of a moderated mediating effect.

Model	COVID-19 event disruption	Indirect effect			Moderated mediating effect		
		*B*	*SE*	95% CI	*B*	*SE*	95% CI
Career calling → role overload → physical fatigue	Low	–0.012	0.039	[−0.100, 0.055]	0.058	0.025	[0.014, 0.115]
	High	0.096	0.030	[0.040, 0.159]			
Career calling → role overload → mental fatigue	Low	–0.015	0.045	[−0.116, 0.071]	0.071	0.030	[0.019, 0.135]
	High	0.118	0.038	[0.044, 0.191]			
Career calling → role overload → emotional fatigue	Low	–0.014	0.043	[−0.103, 0.066]	0.068	0.028	[0.017, 0.129]
	High	0.112	0.035	[0.045, 0.186]			

## Discussion

Drawing on the JD-R model and EST, this study constructed a moderated mediating model to examine how and when career calling was related to work fatigue among Chinese police officers under the condition of COVID-19 event disruption. The results showed that career calling had a U-shaped curvilinear relationship with physical, mental, and emotional fatigue, and role overload partially mediated these direct relationships. Furthermore, COVID-19 event disruption positively moderated the direct relationship of career calling with role overload, physical fatigue, and emotional fatigue, and positively moderated the first stage of the mediating effects. These findings have important theoretical and practical implications.

### Theoretical Contributions

This study offers four theoretical contributions to the career calling literature. First, the results advance a better theoretical understanding of the curvilinear effect of career calling on detrimental workplace outcomes and advance the research stream on the potential curvilinear effect of career calling. Although previous studies revealed the double-edged-sword effect of career calling on individual and organizational workplace outcomes (Duffy and Dik, 2013; Duffy et al., 2014, 2016, 2018; Hirschi et al., 2019), they reported the effects in a linear rather than a curvilinear way. This study addressed the resulting research gap and actively responded to an appeal for further research on the curvilinear effect of career calling on workplace consequences among various occupational groups (Duffy et al., 2018; Lysova et al., 2019). We found that career calling had a U-shaped curvilinear relationship with work fatigue and role overload. Under the framework of the JD-R model (Demerouti et al., 2001), a moderate level of career calling will heighten job-related personal resources appropriately, which is efficient for balancing job demands. Thus, the positive nature of career calling will decrease work fatigue and role overload without resource loss or depletion. In contrast, an excessively high level of career calling will aggravate the burden of social responsibility and career norms placed on police officers when faced with excessive job demands. In this situation, constant demands increasing will simultaneously lead to excessive resource depletion, resulting in detrimental workplace outcomes.

Second, by constructing a moderated mediating model to examine the mediating effect of role overload and the moderating effect of COVID-19 event disruption, this study enriches the present theoretical framework of career calling and provides new directions for future research on the mechanism underlying how career calling affects detrimental workplace outcomes. Existing studies have focused on the mechanism underlying the effect of career calling on workplace outcomes, for instance, the mediating role of career adaptability (Xie et al., 2016), work role fit, psychological meaningfulness (Rothmann and Hamukang’andu, 2013), occupational identity clarity, and occupational self-efficacy (Hirschi, 2012) between career calling and work engagement; affective commitment between career calling and in-role performance (Kim et al., 2018); work meaning between career calling and work positive work attitudes (Steger et al., 2010); and organizational instrumentality between career calling and organization-directed citizenship behavior and job satisfaction (Xie et al., 2017). However, most studies adopted a positive perspective in a linear way. This study took a negative perspective and unpacked the “black box” in the U-shaped curvilinear relationship between career calling and work fatigue by examining the mediating effect of role overload. The results showed that role overload is a partial mediator in the relationship between career calling and work fatigue. This is in line with the JD-R model, which asserts that resource loss and depletion accompanied by serious role overload will further lead to work fatigue. Thus, the results highlight the importance of role overload in the curvilinear effect of career calling on work fatigue.

Third, this study deepens our knowledge about how a global major emergency like the COVID-19 pandemic can affect the relationship between career calling and negative outcomes. EST asserts that workplace or social events can influence individuals’ mental status and behavior patterns from top to bottom (Morgeson et al., 2015; Chen et al., 2020). We provided empirical support for how such a disruptive event affects the mechanism underlying the effect of career calling on work fatigue via role overload. The results showed that COVID-19 event disruption positively moderated the direct effect of career calling on role overload, physical fatigue, and emotional fatigue, respectively, and the first stage of the mediating effect. When COVID-19 event disruption was high, the relationships were stronger. According to EST, the COVID-19 pandemic has had global disruptive effects (Cunill et al., 2020; Zheng, 2020). This is particularly true for police officers, with disruptions affecting their physical and mental health, manifesting in such symptoms as insomnia, stress, and fatigue (Fu et al., 2020; Laufs and Waseem, 2020; Zhu et al., 2020). Under high COVID-19 event disruption, officers perceived an exaggerated level of career calling stemming from social responsibility, and the inherent meaning associated with their identity aggravated their work fatigue by increasing their workload and role load. These results expand previous studies that focused on micro individual factors, rather than macro social factors (Duffy et al., 2018; Huang et al., 2018; Lysova et al., 2019). Moreover, this study expands our knowledge about the relationship between career calling and negative outcomes in the latest theoretical model of career calling (the *work as a calling* theory) (Duffy et al., 2018).

Fourth, by focusing on police officers as a special occupational group in Chinese culture, this study identified the different effects of career calling among emergency occupational groups in this particular culture and context (Duffy et al., 2018; Lysova et al., 2019). Previous studies explored the relationship between career calling in college students, workers, priests, and zookeepers, but few have focused on police officers, particularly Chinese police officers, who are members of a collectivistic culture and base their career calling on community and social needs to a higher extent (Dik and Duffy, 2015). By concentrating on the police occupational group and systematically examining the curvilinear effect of career calling on work fatigue, this study enriches the body of knowledge from previous research and paves the way for future studies on career calling and other workplace outcomes in different cultures and occupational groups to address issues of generalizability (Thompson and Bunderson, 2019).

### Practical Implications

Our findings also have several valuable practical implications. First, the curvilinear effect of career calling on detrimental workplace outcomes among police officers is important for organizations and individuals. Our results showed that career calling had a direct curvilinear effect on work fatigue and an indirect effect via role overload by increasing and depleting job-related personal resources. Thus, organizations should be aware of such curvilinear effect of career calling on police officers’ role overload and work fatigue and appropriately assign tasks to ensure that job demands are fit for officers’ available resources, as this may reduce role overload and work fatigue. In addition, police officers themselves should also adjust their level of career calling to keep job demands and personal resources balanced. A moderate rather than an excessive level of career calling should be encouraged at work, especially in training programs. As seen, beyond an optimal point, the positive effect of career calling on work fatigue and role overload can become negative.

The results also shed light on the moderating role of COVID-19 event disruption, showing that a high COVID-19 event disruption both strengthened the direct U-shaped curvilinear relationship between career calling and role overload, physical fatigue, and emotional fatigue, and the first stage of the mediating effect of role overload. It is well known that the COVID-19 pandemic has had a more significant effect on police officers’ physical and psychological health in terms of chronic stress, high risks, role overload, and workload than on civilians (Zhu et al., 2020). Hence, before such a major public emergency, organizations should prepare emergency plans and carry out exercises to increase police officers’ fitness and minimize their role overload and work fatigue during the emergency period.

Finally, more organizational and family support should be provided, because social resources such as organizational and family support may have beneficial workplace outcomes, such as promoting fitness and increasing work and life satisfaction, positive emotion, and commitment (Duffy et al., 2018). Moreover, some police officers experienced physical and psychological symptoms, including sleep problems, compulsion, and depression, related to role overload, workload, and risks during the COVID-19 pandemic (Teng et al., 2020; Zhu et al., 2020). Therefore, after a significant disruptive event such as the COVID-19 pandemic, first responders should be allowed self-adjustment time to relax, family relationship assistance through telephone/internet, and applied interventions to reduce family conflicts (Stankovska et al., 2020; Teng et al., 2020), which are services that most police officers requested (Zhu et al., 2020).

### Limitations and Future Research

This study has several limitations. First, it used a cross-sectional design and self-report measures; thus, a degree of common method bias was inevitable, and causal relationships between career calling, work fatigue, role overload, and event disruption could not be inferred (Podsakoff et al., 2003). Thus, experimental, time-lag, and longitudinal designs are encouraged in future research. Second, this study only examined the curvilinear relationship between career calling and work fatigue via role overload in the context of COVID-19 event disruption. Future studies should investigate potential negative curvilinear effects of career calling on individual and organizational workplace outcomes, particularly for first responders to major emergencies, including healthcare workers and community volunteers. Finally, we regarded the COVID-19 pandemic disruption as an important contextual factor to explore its effect on the mechanism underlying the effect of career calling on work fatigue. To deeply understand the effect of the COVID-19 pandemic, the boundary effect of the COVID-19 pandemic criticality and novelty should be further examined and synergistically grounded in EST.

## Data Availability Statement

The datasets generated for this study are available on request to the corresponding author.

## Ethics Statement

The studies involving human participants were reviewed and approved by Sichuan Police College Ethics Committee. The patients/participants provided their written informed consent to participate in this study.

## Author Contributions

JZ designed, performed, analyzed, and completed the manuscript. JWZ designed this research. XX assisted in revision.

## Conflict of Interest

The authors declare that the research was conducted in the absence of any commercial or financial relationships that could be construed as a potential conflict of interest.

## References

[B1] Aronsson G. Theorell T. Grape T. Hammarström A. Hogstedt C. Marteinsdottir I. (2017). A systematic review including meta-analysis of work environment and burnout symptoms. *BMC Public Health* 17:264–277. 10.1186/s12889-017-4153-7 28302088 PMC5356239

[B2] Bacharach S. B. Bamberger P. A. (2007). 9/11 and New York city firefighters’ post hoc unit support and control climates: a context theory of the consequences of involvement in traumatic work-related events. *Acad. Manage. J.* 50 849–868. 10.5465/amj.2007.26279180

[B3] Berkelaar B. L. Buzzanell P. M. (2015). Bait and switch or double-edged sword? The (sometimes) failed promises of calling. *Hum. Relat.* 68 157–178. 10.1177/0018726714526265

[B4] Bin A. A. H. (2008). Perceptions of farm stressors among New Zealand farm managers. *Labour, Employment and Work in New Zealand* s1 252–258. 10.26686/lew.v0i0.1637

[B5] Bolino M. C. Turnley W. H. (2005). The personal costs of citizenship behavior: the relationship between individual initiative and role overload, job stress, and work-family conflict. *J. Appl. Psychol.* 90 740–748. 10.1037/0021-9010.90.4.740 16060790

[B6] Bowling N. A. Alarcon G. M. Bragg C. B. Hartman M. J. (2015). A meta-analytic examination of the potential correlates and consequences of workload. *Work Stress* 29 95–113. 10.1080/02678373.2015.1033037

[B7] Brislin R. W. (1980). “Translation and content analysis of oral and written material,” in *Handbook of Cross-Cultural Psychology*, eds Triandis H. C. Berry J. W. (Boston, MA: Allyn and Bacon).

[B8] Brooks S. K. Webster R. K. Smith L. E. Woodland L. Wessely S. Greenberg N. (2020). The psychological impact of quarantine and how to reduce it: rapid review of the evidence. *Lancet* 395 912–920. 10.1016/s0140-6736(20)30460-8 32112714 PMC7158942

[B9] Bunderson J. S. Thompson J. A. (2009). The call of the wild: zookeepers, callings, and the double-edged sword of deeply meaningful work. *Adm. Sci. Q.* 54 32–57. 10.2189/asqu.2009.54.1.32 21821037

[B10] Cardador M. T. Caza B. B. (2012). Relational and identity perspectives on healthy versus unhealthy pursuit of callings. *J. Career Assess.* 20 338–353. 10.1177/1069072711436162

[B11] Chen J. May D. R. Schwoerer C. E. Augelli B. (2016). Exploring the boundaries of career calling. *J. Career Dev.* 45 103–116. 10.1177/0894845316671214

[B12] Chen Y. Liu D. Tang G. Hogan T. M. (2020). Workplace events and employee creativity: a multi-study field investigation. *Pers. Psychol.* 10.1111/peps.12399 [Epub ahead of print].

[B13] Clinton M. E. Conway N. Sturges J. (2017). “It’s tough hanging-up a call”: the relationships between calling and work hours, psychological detachment, sleep quality, and morning vigor. *J. Occup. Health Psychol.* 22 28–39. 10.1037/ocp0000025 27054502

[B14] Cohen L. Duberley J. (2015). Three faces of context and their implications for career: a study of public sector careers cut short. *J. Vocat. Behav.* 91 189–202. 10.1016/j.jvb.2015.10.006

[B15] Coverman S. (1989). Role overload, role conflict, and stress: addressing consequences of multiple role demands. *Soc. Forces* 67 965–982. 10.2307/2579710

[B16] Creed P. A. Rogers M. E. Praskova A. Searle J. (2014). Career calling as a personal resource moderator between environmental demands and burnout in Australian junior doctors. *J. Career Dev.* 41 547–561. 10.1177/0894845313520493

[B17] Cunill M. Aymerich M. Serdà B. Patiño-Masó J. (2020). The impact of COVID-19 on Spanish health professionals: a description of physical and psychological effects. *Int. J. Ment. Health Promot.* 22 185–198. 10.32604/IJMHP.2020.011615

[B18] Dasgupta P. (2012). Effect of role ambiguity, conflict and overload in private hospitals’ nurses’ burnout and mediation through self-efficacy. *J. Health Manage.* 14 513–534. 10.1177/0972063412468980

[B19] Daskin M. (2016). Linking polychronicity to hotel frontline employees’ job outcomes. *Euromed J. Bus.* 11 162–180. 10.1108/EMJB-04-2015-0022

[B20] De Beer L. T. Pienaar J. Rothmann S. (2015). Work overload, burnout, and psychological ill-health symptoms: a three-wave mediation model of the employee health impairment process. *Anxiety Stress Coping* 29 387–399. 10.1080/10615806.2015.1061123 26079200

[B21] Demerouti E. Bakker A. B. Nachreiner F. Schaufeli W. B. (2001). The job demands-resources model of burnout. *J. Appl. Psychol.* 86 499–512. 10.1037/0021-9010.86.3.499 11419809

[B22] Deng Y. X. Chen X. P. (2019). Relationship between professional mission of grassroots police and job burnout: the regulating role of psychological disengagement. *Chin. J. Health Psychol.* 27 1394–1399. 10.13342/j.cnki.cjhp.2019.09.029

[B23] Dik B. J. Duffy R. D. (2012). *Make Your Job a Calling: How the Psychology of Vocation Can Change Your Life at Work.* Philadelphia, PA: Templeton Press.

[B24] Dik B. J. Duffy R. D. (2015). “Strategies for discerning and living a calling,” in *APA Handbook of Career Intervention*, eds Hartung P. Savickas M. Walsh B. (Washington, DC: American Psychological Association), 305–317. 10.1037/14439-023

[B25] Ding H. Yu E. H. (2020). Followers’ strengths-based leadership and strengths use of followers: the roles of trait emotional intelligence and role overload. *Pers. Individ. Differ.* 168:110300 10.1016/j.paid.2020.110300

[B26] Dobrow S. R. (2013). Dynamics of calling: a longitudinal study of musicians. *J. Organ. Behav.* 34 431–452. 10.1002/job.1808

[B27] Dobrow S. R. Tosti-Kharas J. (2011). Calling: the development of a scale measure. *Pers. Psychol.* 64 1001–1049. 10.1111/j.1744-6570.2011.01234.x

[B28] Douglass R. P. Duffy R. D. Autin K. L. (2015). Living a calling, nationality, and life Satisfaction. *J. Career Assess.* 24 253–269. 10.1177/1069072715580324

[B29] Duffy R. D. Allan B. A. Autin K. L. Bott E. M. (2013). Calling and life satisfaction: it’s not about having it, it’s about living it. *J. Couns. Psychol.* 60 42–52. 10.1037/a0030635 23163611

[B30] Duffy R. D. Allan B. A. Bott E. M. Dik B. J. (2014). Does the source of a calling matter? External summons, destiny, and perfect fit. *J. Career Assess.* 22 562–574. 10.1177/1069072713514812

[B31] Duffy R. D. Bott E. M. Allan B. A. Torrey C. L. Dik B. J. (2012). Perceiving a calling, living a calling, and job satisfaction: testing a moderated, multiple mediator model. *J. Couns. Psychol.* 59 50–59. 10.1037/a0026129 22059426

[B32] Duffy R. D. Dik B. J. (2013). Research of calling: What have we learned and where are we going? *J. Vocat. Behav.* 83 428–436. 10.1016/j.jvb.2013.06.006

[B33] Duffy R. D. Dik B. J. Douglass R. P. England J. W. Velez B. L. (2018). Work as a calling: a theoretical model. *J. Couns. Psychol.* 65 423–439. 10.1037/cou0000276 29999369

[B34] Duffy R. D. Douglass R. P. Autin K. L. England J. Dik B. J. (2016). Does the dark side of a calling exist? Examining potential negative effects. *J. Posit. Psychol.* 11 634–646. 10.1080/17439760.2015.1137626

[B35] Duxbury L. Higgins C. Halinski M. (2015). Identifying the antecedents of work-role overload in police organizations. *Crim. Justice Behav.* 42 361–381. 10.1177/0093854814551017

[B36] Eisenberger R. Huntington R. Hutchison S. Sowa D. (1986). Perceived organizational support. *J. Appl. Psychol.* 71 500–507. 10.1037/0021-9010.71.3.500

[B37] Elangovan A. R. Pinder C. C. McLean M. (2010). Callings and organizational behavior. *J. Vocat. Behav.* 76 428–440. 10.1016/j.jvb.2009.10.009

[B38] Fekedulegn D. Burchfiel C. M. Ma C. C. Andrew M. E. Hartley T. A. Charles L. E. (2017). Fatigue and on-duty injury among police officers: the BCOPS study. *J. Saf. Res.* 60 43–51. 10.1016/j.jsr.2016.11.006 28160813 PMC6311701

[B39] Frone M. R. Tidwell M. C. (2015). The meaning and measurement of work fatigue: development and evaluation of the three-dimensional work fatigue inventory (3D-WFI). *J. Occup. Health Psychol.* 20 273–288. 10.1037/a0038700 25602275 PMC4505929

[B40] Fu W. N. Wang C. Zou L. Guo Y. Y. Lu Z. X. Yan S. J. (2020). Psychological health, sleep quality, and coping styles to stress facing the COVID-19 in Wuhan, China. *Transl. Psychiatry* 10 225–234. 10.1038/s41398-020-00913-3 32647160 PMC7347261

[B41] Hagmaier T. Abele A. E. (2012). The multidimensionality of calling: conceptualization, measurement and a bicultural perspective. *J. Vocat. Behav.* 81 39–51. 10.1016/j.jvb.2012.04.001

[B42] Hagmaier T. Abele A. E. (2015). When reality meets ideal. *J. Career Assess.* 23 367–382. 10.1177/1069072714547164

[B43] Hall D. T. Chandler D. E. (2005). Psychological success: when the career is a calling. *J. Organ. Behav.* 26 155–176. 10.1002/job.301

[B44] Hayes A. F. (2013). *Introduction to Mediation, Moderation, and Conditional Process Analysis: A Regression-Based Approach.* New York, NY: The Guilford Press.

[B45] Hecht L. M. (2001). Role conflict and role overload: different concepts, different consequences. *Sociol. Inq.* 71 111–121. 10.1111/j.1475-682x.2001.tb00930.x

[B46] Hirschi A. (2011). Callings in career: a typological approach to essential and optional components. *J. Vocat. Behav.* 79 60–73. 10.1016/j.jvb.2010.11.002

[B47] Hirschi A. (2012). Callings and work engagement: moderated mediation model of work meaningfulness, occupational identity, and occupational self-efficacy. *J. Couns. Psychol.* 59 479–485. 10.1037/a0028949 22774870

[B48] Hirschi A. Herrmann A. (2012). Vocational identity achievement as a mediator of presence of calling and life satisfaction. *J. Career Assess.* 20 309–321. 10.1177/1069072711436158

[B49] Hirschi A. Keller A. C. Spurk D. M. (2019). Calling as a double-edged sword for work-nonwork enrichment and conflict among older workers. *J. Vocat. Behav.* 114 100–111. 10.1016/j.jvb.2019.02.004

[B50] Huang L. Xie L. X. Ding S. Q. (2018). Review and prospect of the sense of professional mission. *Contemp. Econ. Manage.* 40 52–60. 10.13253/j.cnki.ddjjgl.2018.08.010

[B51] Johns G. (2017). Reflections on the 2016 decade award: incorporating context in organizational research. *Acad. Manage. Rev.* 42 577–595. 10.5465/amr.2017.0044

[B52] Keller A. C. Spurk D. Baumeler F. Hirschi A. (2016). Competitive climate and workaholism: negative sides of future orientation and calling. *Pers. Individ. Differ.* 96 122–126. 10.1016/j.paid.2016.02.061

[B53] Kim C. Lee J. Shin S. (2019). Why are your employees leaving the organization? The interaction effect of role overload, perceived organizational support, and equity sensitivity. *Sustainability* 11:657 10.3390/su11030657

[B54] Kim S. S. Shin D. Vough H. C. Hewlin P. F. Vandenberghe C. (2018). How do callings relate to job performance? The role of organizational commitment and ideological contract fulfillment. *Hum. Relat.* 71 1319–1347. 10.1177/0018726717743310

[B55] Kristie B. (2017). *Role Overload and Job Stress: The Role of Perceived Organizational Support.* Bachelor’s thesis, Tilburg University, Tilburg.

[B56] Laufs J. Waseem Z. (2020). Policing in pandemics: a systematic review and best practices for police response to COVID-19. *Int. J. Disaster Risk Reduct.* 51:101812. 10.1016/j.ijdrr.2020.101812 32839687 PMC7439012

[B57] Li Q. Wang H. Y. (2018). Role overload in organizations. *Adv. Psychol. Sci.* 26 2046–2056. 10.3724/SP.J.1042.2018.02046

[B58] Liu D. Liu J. (2017). Dissecting event system theory: tenets and opportunities for research and practice. *Q. J. Manage.* 2 64–80.

[B59] Lysova E. I. Dik B. Duffy R. D. Khapova S. N. Arthur M. B. (2019). Calling and careers: new insights and future directions. *J. Vocat. Behav.* 114 1–6. 10.1016/j.jvb.2019.03.004

[B60] Lysova E. I. Jansen P. G. Khapova S. N. Plomp J. Tims M. (2018). Examining calling as a double-edged sword for employability. *J. Vocat. Behav.* 104 261–272. 10.1016/j.jvb.2017.11.006

[B61] Mainemelis C. (2001). When the muse takes it all: a model for the experience of timelessness in organizations. *Acad. Manage. Rev.* 26 548–565. 10.5465/AMR.2001.5393891

[B62] Michaelson C. Tosti-Kharas J. (2019). Serving self or serving others? Close relations’ perspectives on ethics and calling. *J. Vocat. Behav.* 114 19–30. 10.1016/j.jvb.2019.02.005

[B63] Morgeson F. P. (2005). The external leadership of self-managing teams: intervening in the context of novel and disruptive events. *J. Appl. Psychol.* 90 497–508. 10.1037/0021-9010.90.3.497 15910145

[B64] Morgeson F. P. DeRue D. S. (2006). Event criticality, urgency, and duration: understanding how events disrupt teams and influence team leader intervention. *Leadersh. Q.* 17 271–287. 10.1016/j.leaqua.2006.02.006

[B65] Morgeson F. P. Mitchell T. R. Liu D. (2015). Event system theory: an event-oriented approach to the organizational sciences. *Acad. Manage. Rev.* 40 515–537.

[B66] Neubert M. J. Halbesleben K. (2015). Called to commitment: an examination of relationships between spiritual calling, job satisfaction, and organizational commitment. *J. Bus. Ethics* 132 859–872. 10.1007/s10551-014-2336-z

[B67] Nixon A. E. Mazzola J. J. Bauer J. Krueger J. R. Spector P. E. (2011). Can work make you sick? A meta-analysis of the relationships between job stressors and physical symptoms. *Work Stress* 25 1–22. 10.1080/02678373.2011.569175

[B68] Örtqvist D. Wincent J. (2006). Prominent consequences of role stress: a meta-analytic review. *Int. J. Stress Manage.* 13 399–422. 10.1037/1072-5245.13.4.399

[B69] Park J. Lee K. Lim J. I. Sohn Y. W. (2018). Leading with callings: effects of leader’s calling on followers’ team commitment, voice behavior, and job performance. *Front. Psychol.* 9:1706. 10.3389/fpsyg.2018.01706 30258386 PMC6143684

[B70] Park J. Sohn Y. W. Ha Y. J. (2015). South Korean salespersons’ calling, job performance, and organizational citizenship behavior. *J. Career Assess.* 24 415–428. 10.1177/1069072715599354

[B71] Pei Y. J. Zhao S. M. (2015). Study on knowledge worker’s calling, career commitment and job attitudes. *J. Manage. Sci.* 28 103–114. 10.3969/j.issn.1672-0334.2015.02.010

[B72] Penney L. M. Hunter E. M. Perry S. J. (2011). Personality and counterproductive work behaviour: using conservation of resources theory to narrow the profile of deviant employees. *J. Occup. Organ. Psychol.* 84 58–77. 10.1111/j.2044-8325.2010.02007.x

[B73] Peterson M. F. Smith P. B. Akande A. Ayestaran S. Bochner S. Callan V. (1995). Role conflict, ambiguity, and overload: a 21-nation study. *Acad. Manage. J.* 38 429–452. 10.2307/256687

[B74] Podsakoff P. M. MacKenzie S. B. Lee J. Y. Podsakoff N. P. (2003). Common method biases in behavioral research: a critical review of the literature and recommended remedies. *J. Appl. Psychol.* 88 879–903. 10.1037/0021-9010.88.5.879 14516251

[B75] Posig M. Kickul J. (2003). Extending our understanding of burnout: test of an integrated model in nonservice occupations. *J. Occup. Health Psychol.* 8 3–19. 10.1037/1076-8998.8.1.3 12553526

[B76] Praskova A. Hood M. Creed P. A. (2014). Testing a calling model of psychological career success in Australian young adults: a longitudinal study. *J. Vocat. Behav.* 85 125–135. 10.1016/j.jvb.2014.04.004

[B77] Presbitero A. Teng-Calleja M. (2019). Employee intention to stay in an organization: examining the role of calling and perceived supervisor support through the theoretical lens of work as calling. *J. Career Assess.* 28 320–336. 10.1177/1069072719858389

[B78] Qiu T. Yang Y. Liu C. Tian F. Gu Z. Yang S. (2020). The association between resilience, perceived organizational support and fatigue among Chinese doctors: a cross-sectional study. *J. Affect. Disord.* 265 85–90. 10.1016/j.jad.2020.01.056 31957696

[B79] Qureshi H. Lambert E. G. Frank J. (2019). The relationship between stressors and police job involvement. *Int. J. Police Sci. Manage.* 2 48–61. 10.1177/1461355719832621

[B80] Ricci J. A. Chee E. Lorandeau A. L. Berger J. (2007). Fatigue in the U.S. workforce: prevalence and implications for lost productive work time. *J. Occup. Environ. Med.* 49 1–10. 10.1097/01.jom.0000249782.60321.2a 17215708

[B81] Rose D. M. Seidler A. Nübling M. Latza U. Brähler E. Klein E. M. (2017). Associations of fatigue to work-related stress, mental and physical health in an employed community sample. *BMC Psychiatry* 17:167. 10.1186/s12888-017-1237-y 28476149 PMC5420158

[B82] Rothmann S. Hamukang’andu L. (2013). Callings, work role fit, psychological meaningfulness and work engagement among teachers in Zambia. *S. Afr. J. Educ.* 33 1–16. 10.15700/saje.v33n2a699

[B83] Schaubroeck J. Cotton J. L. Jennings K. R. (1989). Antecedents and consequences of role stress: a covariance structure analysis. *J. Organ. Behav.* 10 35–58. 10.1002/job.4030100104

[B84] Stankovska G. Memedi I. Dimitrovski D. (2020). Coronavirus COVID-19 disease, mental health and psychological support. *Soc. Reg.* 4 33–48. 10.14746/sr.2020.4.2.03

[B85] Steger M. F. Pickering N. K. Shin J. Y. Dik B. J. (2010). Calling in work: Secular or sacred? *J. Career Assess.* 18 82–96. 10.1177/1069072709350905

[B86] Stogner J. Miller B. L. McLean K. (2020). Police stress, mental health, and resiliency during the COVID-19 pandemic. *Am. J. Crim. Justice* 41 718–730. 10.1007/s12103-020-09548-y 32837167 PMC7319488

[B87] Sturges J. Clinton M. Conway N. Budjanovcanin A. (2019). I know where I’m going: sensemaking and the emergence of calling. *J. Vocat. Behav.* 114 57–68. 10.1016/j.jvb.2019.02.006

[B88] Suarthana J. H. P. Riana I. G. (2016). The effect of psychological contract breach and workload on intention to leave: mediating role of job stress. *Procedia Soc. Behav. Sci.* 219 717–723. 10.1016/j.sbspro.2016.05.056

[B89] Surana S. J. Singh A. K. (2013). The impact of role stressors and work overload on job burnout. *Int. J. Intell. Enterp.* 2 64–83. 10.1504/ijie.2013.057339

[B90] Teng Z. Wei Z. Qiu Y. Tan Y. Chen J. Tang H. (2020). Psychological status and fatigue of frontline staff two months after the COVID-19 pandemic outbreak in China: a cross-sectional study. *J. Affect. Disord.* 275 247–252. 10.1016/j.jad.2020.06.032 32734915 PMC7330556

[B91] Thompson J. A. Bunderson J. S. (2019). Research on work as a calling…and how to make it matter. *Annu. Rev. Organ. Behav.* 6 421–443. 10.1146/annurev-orgpsych-012218-015140

[B92] Van Iddekinge C. H. Aguinis H. Mackey J. D. De Ortentiis P. S. (2018). A meta-analysis of the interactive, additive, and relative effects of cognitive ability and motivation on performance. *J. Manag.* 44 249–279. 10.1177/0149206317702220

[B93] Vullinghs J. T. De Hoogh A. H. B. Den Hartog D. N. Boon C. (2018). Ethical and passive leadership and their joint relationships with burnout via role clarity and role overload. *J. Bus. Ethics* 165 1–15. 10.1007/s10551-018-4084-y

[B94] Wright T. A. Cropanzano R. (2000). Psychological well-being and job satisfaction as predictors of job performance. *J. Occup. Health Psychol.* 5 84–94. 10.1037/1076-8998.5.1.84 10658888

[B95] Xanthopoulou D. Bakker A. B. Demerouti E. Schaufeli W. B. (2009). Reciprocal relationships between job resources, personal resources, and work engagement. *J. Vocat. Behav.* 74 235–244. 10.1016/j.jvb.2008.11.003

[B96] Xiang Y. T. Yang Y. Li W. Zhang L. Zhang Q. Cheung T. (2020). Timely mental health care for the 2019 novel coronavirus outbreak is urgently needed. *Lancet Psychiatry* 7 228–229. 32032543 10.1016/S2215-0366(20)30046-8 PMC7128153

[B97] Xie B. Zhou W. Huang J. L. Xia M. (2017). Using goal facilitation theory to explain the relationships between calling and organization-directed citizenship behavior and job satisfaction. *J. Vocat. Behav.* 100 78–87. 10.1016/j.jvb.2017.03.001

[B98] Xie B. G. Xia M. Xin X. Zhou W. X. (2016). Linking calling to work engagement and subjective career success: the perspective of career construction theory. *J. Vocat. Behav.* 94 70–78. 10.1016/j.jvb.2016.02.011

[B99] Zhang C. Y. Dik B. J. Wei J. Zhang J. F. (2014). Work as a calling in China. *J. Career Assess.* 23 236–249. 10.1177/1069072714535029

[B100] Zhang C. Y. Herrmann A. Hirschi A. Wei J. Zhang J. F. (2015). Assessing calling in Chinese college students. *J. Career Assess.* 23 582–596. 10.1177/1069072715595804

[B101] Zhang C. Y. Hirschi A. Herrmann A. Wei J. Zhang J. F. (2017). The future work self and calling: the mediational role of life meaning. *J. Happiness Stud.* 18 977–991. 10.1007/s10902-016-9760-y

[B102] Zhao X. Y. Xue G. Y. Guo C. (2016). Relationship of sense of calling to workload and job satisfaction in coal enterprise employee. *Chin. Ment. Health J.* 30 64–69. 10.3969/j.issn.1000-6729.2016.01.013

[B103] Zheng J. H. (2020). “Watch” or “participate”: the mediating effect of the attributes of COVID-19 between spatial distance and social participation. *J. East Chin. UniV. Sci. Technol.* 35 100–112.

[B104] Zhu X. Xia M. Hu Y. Zhang L. Lu Y. Zhang Y. (2020). Mental status and psychological needs of Chinese police officers in a highly impacted city during the COVID-19 pandemic. *Int. J. Ment. Health Promot.* 22 149–157. 10.32604/IJMHP.2020.011097

